# The Impacts of Reassortant Avian Influenza H5N2 Virus NS1 Proteins on Viral Compatibility and Regulation of Immune Responses

**DOI:** 10.3389/fmicb.2020.00280

**Published:** 2020-03-12

**Authors:** Wen-Chien Wang, Chih-Ying Kuan, Yu-Jing Tseng, Chia-Hsuan Chang, Yee-Chen Liu, Yu-Chih Chang, Yu-Chen Hsu, Ming-Kun Hsieh, Shan-Chia Ou, Wei-Li Hsu

**Affiliations:** Graduate Institute of Microbiology and Public Health, College of Veterinary Medicine, National Chung Hsing University, Taichung, Taiwan

**Keywords:** avian influenza virus, H5N2, NS1, reverse genetics, RNA-dependent RNA polymerase activity

## Abstract

Avian influenza virus (AIV) can cause severe diseases in poultry worldwide. H6N1 AIV was the dominant enzootic subtype in 1985 in the chicken farms of Taiwan until the initial outbreak of a low pathogenic avian influenza (LPAI) H5N2 virus in 2003; thereafter, this and other LPAIs have been sporadically detected. In 2015, the outbreak of three novel H5Nx viruses of highly pathogenic avian influenza (HPAI) emerged and devastated Taiwanese chicken and waterfowl industries. The mechanism of variation in pathogenicity among these viruses is unclear; but, in light of the many biological functions of viral non-structural protein 1 (NS1), including interferon (IFN) antagonist and host range determinant, we hypothesized that NS genetic diversity contributes to AIV pathogenesis. To determine the impact of NS1 variants on viral infection dynamics, we established a reverse genetics system with the genetic backbone of the enzootic Taiwanese H6N1 for generation of reassortant AIVs carrying exogenous NS segments of three different Taiwanese H5N2 strains. We observed distinct cellular distributions of NS1 among the reassortant viruses. Moreover, exchange of the NS segment significantly influenced growth kinetics and induction of cytokines [IFN-α, IFN-β, and tumor necrosis factor alpha (TNF-α)] in an NS1- and host-specific manner. The impact of NS1 variants on viral replication appears related to their synergic effects on viral RNA-dependent RNA polymerase activity and IFN response. With these approaches, we revealed that NS1 is a key factor responsible for the diverse characteristics of AIVs in Taiwan.

## Introduction

Avian influenza virus (AIV) causes highly contagious diseases in poultry worldwide and poses a severe public health threat to humans (Shi et al., 2016). For the last two decades, H6N1 has been the enzootic AIV subtype in Taiwanese chicken farms (Lu et al., 1985; Lee et al., 2006, 2014), until an outbreak of low pathogenic avian influenza (LPAI) H5N2 occurred in 2003, followed by a second wave in 2008 (Cheng et al., 2010). This H5N2 was a reassortant virus, with hemagglutinin (HA) and neuraminidase (NA) likely derived from a Mexican-like H5N2 and internal genes from the enzootic H6N1 lineage. The Taiwanese H5N2 evolved to a highly pathogenic avian influenza (HPAI) in 2012; sequence analysis indicated that these H5N2 viruses harbor multiple basic amino acids at the cleavage site of the HA-connecting peptides and that they co-circulate with H6N1 (Lee et al., 2014). In early 2015, three novel H5Nx viruses (H5N2, H5N3, and H5N8, all of which belong to Asian HPAI H5 clade 2.3.4.4) emerged in Taiwan (Huang et al., 2016). These H5Nx viruses mainly affected geese, where they caused high mortality (Lee et al., 2016). At present, LPAI virus (LPAIV) H6N1 and different pathogenic H5 viruses are the major subtypes circulating in local poultry in Taiwan.

The genome of AIV is composed of eight viral RNA (vRNA) segments (McGeoch et al., 1976). The non-structural (NS) segment encodes NS protein 1 (NS1) and NS2 [also known as nuclear export protein (NEP)], which are translated from spliced NS transcripts (Palese and Shaw, 2007; Dubois et al., 2014; Kuo et al., 2016a). NS1 is a multifunctional protein that impacts both viral infection and cellular biosynthesis (Ayllon and Garcia-Sastre, 2015). Extensive studies demonstrate its major role in modulation of host anti-viral response and, in particular, on counteracting interferon (IFN) (García-Sastre, 2001; Seo et al., 2002; Hale et al., 2008). NS1 binds double-stranded RNA (dsRNA) and interacts with protein kinase R (PKR) in a manner that ultimately leads to suppression of PKR activation (Min and Krug, 2006). Moreover, NS1 interacts with the 30-kDa cleavage and polyadenylation specificity factor (F30) and inhibits the 3′ processing of IFN-α/β pre-mRNA (Noah et al., 2003; Twu et al., 2006). Previous studies have also demonstrated that exchange of NS segments affects viral replication, pathogenicity, and host adaptation in HPAI viruses (HPAIVs) (Ma et al., 2010); and deletion of NS1 attenuates the virulence of H5N1 viruses (Steel et al., 2009; Shi et al., 2016). Thus, NS1 plays a critical role in host adaptation and pathogenesis of influenza viruses.

In light of the diversity of AIVs in recent outbreaks, NS variation may play a pivotal role in the distinct characteristics of AIVs independent from the new HA and NA subtypes. Previous studies of avian influenza in Taiwan have mainly focused on epidemiology, and little is known about the contribution of individual protein function on virulence or host adaptation. To investigate the impact of NS1, we engineered reassortant viruses with the NS segment derived from contemporary Taiwanese H5 strains in the backbone of an enzootic H6N1 virus by reverse genetics. Of the three local H5N2 viruses selected for this study, two viruses, despite different pathogenicity, contain internal genes derived from H6N1 lineage, whereas one HPAIV consists of internal genes of a variety of phylogeny and with unusual virulence in waterfowl (Lee et al., 2014, 2016). In the current study, substitution of a single genome segment would define the contribution of NS, particularly to viral replication, and cytokine induction which will be evaluated in different *in vitro* and *in vivo* models. The results demonstrated that NS gene substitution markedly affects viral growth kinetics and host cytokine expression in avian and mammalian cells.

## Materials and Methods

### Virus and Cells

H6N1 viruses (A/chicken/Taiwan/0702/2013) were propagated in 10-day-old embryonated specific-pathogen-free (SPF) chicken eggs for 72 h at 37°C. Madin–Darby canine kidney (MDCK) cells, chicken DF-1 cells, human A549 cells, human embryonic kidney HEK293T cells, and duck embryo (DE) cells were maintained in Dulbecco’s modified Eagle’s medium (DMEM, Gibco BRL, Life Technologies Corporation, Carlsbad, CA, United States) supplemented with 10% fetal bovine serum (FBS; HyClone, Logan, UT, United States) and antibiotics.

### Construction of Plasmids for Reverse Genetics

The RNA of A/chicken/Taiwan/0702/2013 was extracted, and the eight segments of the viral genome were amplified by RT-PCR (primers, Table 1). All primers contained the restriction enzyme *Sap*I and the non-coding regions of each segment. The reverse genetic vector pDZ was kindly provided by Professor Peter Palese (Icahn School of Medicine at Mount Sinai, United States) (Martinez-Sobrido and Garcia-Sastre, 2010). The eight PCR products with sequences of individual gene segments were digested by *Sap*I (New England Biolabs, Beverly, MA, United States) and then cloned into the pDZ vector. Following the same strategy, the NS gene segments of the other three H5N2 AIVs were commercially synthesized by Allbio Science Inc., (Taichung, Taiwan) for generation of the reassortant AIVs, including two Mexican-like strains, A/chicken/Taiwan/683/2012 and A/chicken/Taiwan/1680/2013, and one that belongs to clade 2.3.4.4, A/goose/Taiwan/01031/2015.

**TABLE 1:** Molecular cloning primers.

Primer name	Sequences (5′–3′)^a^	Plasmid
NS-RG-F	TATTGCTCTTCAGGGAGCAAAAGCAGGGTG	NS-pDZ
NS-RG-R	ATATGCTCTTCGTATTAGTAGAAACAAGGGTGTTTT	
M-RG-F	TATTGCTCTTCAGGGAGCAAAAGCAGGTAG	M-pDZ
M-RG-R	ATATGCTCTTCGTATTAGTAGAAACAAGGTAGTTTTT	
NA-RG-F	TATTGCTCTTCAGGGAGCAAAAGCAGGAGT	NA-pDZ
NA-RG-R	ATATGCTCTTCGTATTAGTAGAAACAAGGAGTTTTTT	
NP-RG-F	TATTGCTCTTCAGGGAGCAAAAGCAGGGTA	NP-pDZ
NP-RG-R	ATATGCTCTTCGTATTAGTAGAAACAAGGGTATTTTT	
HA-RG-F	TATTGCTCTTCAGGGAGCAAAAGCAGGGG	HA-pDZ
HA-RG-R	ATATGCTCTTCGTATTAGTAGAAACAAGGGTGTTTT	
PA-RG-F	TATTGCTCTTCAGGGAGCGAAAGCAGGTAC	PA-pDZ
PA-RG-R	ATATGCTCTTCGTATTAGTAGAAACAAGGTACTT	
PB1-RG-F	TATTGCTCTTCAGGGAGCGAAAGCAGGCA	PB1-pDZ
PB1-RG-R	ATATGCTCTTCGTATTAGTAGAAACAAGGCATTT	
PB2-RG-F	TATTGCTCTTCAGGGAGCGAAAGCAGGTC	PB2-pDZ
PB2-RG-R	ATATGCTCTTCGTATTAGTAGAAACAAGGTCGTTT	
NS1-EcoR-F1	AAGAATTCAT*GGATTC*CAACACTGTGTCAAG	NS0702-FLAG NS683-FLAG NS1031-FLAG
NS1-Kpn-R2	TT*GGTACC*TTAACTTCTGGCTCAATTGTTCTC	NS0702-FLAG NS683-FLAG
NS1031-Kpn-R2	TT*GGTACC*TTAACTTCTGACTCAATTGTTCTC	NS1031-FLAG
NS1-EcoR-F2	AA*GAATTC*ATGGATCCAAACACTGTGTCAAG	NS1680-FLAG
NS1680-Kpn-R3	TT*GGTACC*TTTTTTGAAGGGAATGGAGATCCC	
NS1-*Bgl*II-F1	AA*AGATCT*ATGGATTCCAACACTGTGTCAAG	NS0702-eGFP NS683-eGFP NS1031-eGFP
NS1-Not-R1	TT*GCGGCCGC*AACTTCTGGCTCAATTGTTCTC	NS0702-eGFP, NS683-eGFP
NS1031-Not-R	TT*GCGGCCGC*AACTTCTGACTCAATTGTTCTC	NS1031-eGFP
NS1-*Bgl*II-F2	AA*AGATCT*ATGGATCCAAACACTGTGTCAAG	NS1680-eGFP
NS1680-Not-R2	TT*GCGGCCGC*TTTTGAAGGGAATGGAGATCCC	
hPKR-Bam-F	GGAA*GGATCC*ATGGCTGGTGATCTTTCAGC	hPKR-HA
hPKR-Xho-R	GGAA*CTCGAG*ACATGTGTGTCGTTCATTTTTCTC	
NS0702NP-F	AA*GCGGCCGC*ATGGCGTCTCAAGGCACC	NS0702-NP
NS0702NP-R	TT*GGTACC*ATTGTCATACTCCTCTGCATTG	
NS0702PA-F	AA*GCGGCCGC*ATGGAAGACTTTGTGCGAC	NS0702-PA
NS0702PA-R	TT*GGTACC*TTTCAGTGCATGTGTGAGG	
NS0702PB1-F	AA*GCGGCCGC*ATGGATGTCAATCCGACTTTAC	NS0702-PB1
NS0702PB1-R	TT*GGTACC*TTTTTGCCGTCTGAGCTCTTC	
NS0702PB2-F	AA*GCGGCCGC*ATGGAGAGAATAAAAGAATTAAG	NS0702-PB2
NS0702PB2-R	TT*GGTACC*ATTGATGGCCATCCGAATTC	

*^a^Underlined data indicate sequences of restriction enzymes.*

### Rescue of Reassortant Avian Influenza Viruses

HEK293T cells (2 × 10^5^/well, 24-well plate) were transfected with the mixture of pDZ plasmids containing eight gene segments (seven genes of A/chicken/Taiwan/0702/2013 and the NS derived from a different viral strain) by Lipofectamine 2000 Reagent (Invitrogen, Carlsbad, CA, United States). Twenty-four hours after transfection, cells were harvested and overlaid onto MDCK cells for amplification of the progeny viruses. Three single plaques of reassortant viruses were isolated and further propagated in SPF eggs. Genome sequences of the purified viruses were validated by automated sequencing.

### Generation of Constructs Expressing Non-structural Protein 1, Viral RNA-Dependent RNA Polymerase Complex, and Protein Kinase R

Two sets of constructs were generated for expression of NS1 proteins with fusion of either a FLAG-tag or enhanced green fluorescent protein (eGFP) at its C-terminus. PCR was used to amplify the coding region of the four NS1 variants. The resulting PCR fragments were subsequently treated with two restriction enzymes, *Eco*RI*/Kpn*I and *Bgl*II/*Not*I, for cloning into vector pCMV14 (Sigma-Aldrich) or GFP-pcDNA3.1 (Tseng et al., 2015), which express FLAG-tag and eGFP, respectively. Human PKR fused with an HA-tag was amplified, treated with *Bam*HI/*Xho*I, and then ligated with vector pcDNA6 linearized with the same set of enzymes (Liao et al., 2019). The components of RNA polymerase (RdRp) complex [PA, PB1, PB2, and nucleoprotein (NP)] in A/chicken/Taiwan/0702/2013, the parental virus, were individually amplified by PCR and cloned into vector pCMV14 (Sigma-Aldrich) linearized with *Kpn*I and *Not*I.

### Plaque Assay

Madin–Darby canine kidney cells seeded in 12-well plates (approximately 90% confluency) were washed with phosphate-buffered saline (PBS) and infected with 400 μl of viruses serially diluted by an infectious medium (DMEM with TPCK-treated trypsin) in 10-fold steps. One hour post adsorption, the infectious medium was removed, and the cells were covered by the infectious medium with 0.6% agarose. At 48 h post infection (hpi), cells were fixed by methanol and stained with crystal violet. Virus titers were calculated and expressed as the mean plaque-forming units (PFUs)/ml.

### Growth Kinetics of Reassortant Avian Influenza Viruses

DF-1, MDCK, and A549 cells were infected with each reassortant virus at a multiplicity of infection (MOI) of 0.01. At 12, 24, 36, and 48 hpi, all the culture media were collected, and the titers were measured by plaque assay.

### Quantification of Cytokine Transcripts With Quantitative Real-Time RT-PCR

The effect of AIVs on cytokine expression was evaluated in cell and animal models. Cell models using avian DF-1 and human A549 cells were infected with reassortant viruses at a MOI of 0.1 in five independent experiments. At 6 and 12 hpi, total RNA was extracted from cells by RNeasy Plus Universal Mini kit (QIAGEN) and processed by TURBO DNA-Free kit (Invitrogen). One-day-old chickens (*n* = 5) were used as an animal model and intratracheally inoculated with 5 × 10^5^ PFU of the reassortant viruses. At 6 and 12 hpi, lungs of the infected animals were collected and homogenized by TissueLyser II (QIAGEN). Total RNA was extracted by Maxwell RSC simplyRNA Tissue kit (Promega). The experimental protocol and sample collection were approved by the Institutional Animal Care and Committee of National Chung Hsing University (IACUC number: 107-148).

Subsequently, cytokine expression was monitored by RT-PCR using gene-specific primers (summarized in Table 2). RNA was reverse-transcribed with GoTaq 1-Step RT-qPCR System kit (Promega) followed by cytokine quantification (CFX Connect Real-Time PCR Detection System, Bio-Rad). Expression data from three independent experiment were relativized by 2^–Δ^ ^Δ^ ^*Ct*^ with endogenous β-actin standard, and all values were estimated as fold above mock group. Moreover, viral M segment was quantified following the method described in one previous report (Spackman and Suarez, 2008).

**TABLE 2:** RT-PCR primers.

Primer name	Sequences (5′–3′)	Amplicon size (bp)	Target gene
huIFN-α-F	GACTCCATCTTGGCTGTGA	103	Human IFN-α
huIFN-α-R	TGATTTCTGCTCTGACAACCT		
huIFN-β-F	GACGCCGCATTGACCATCTA	298	Human IFN-β
huIFN-β-R	CCTTAGGATTTCCACTCTGACT		
huTNF-α-F	CCCAGGGACCTCTCTCTAATC	84	Human TNF-α
huTNF-α-R	ATGGGCTACAGGCTTGTCACT		
hu-actin-F	CTCTTCCAGCCTTCCTTCCT	115	Human β-actin
hu-actin-R	AGCACTGTGTTGGCGTACAG		
ch-IFN-α-F	GACATCCTTCAGCATCTCTTCA	238	Chicken IFN-α
chIFN-α-R	AGGCGCTGTAATCGTTGTCT		
chIFN-β-F	CCTCAACCAGATCCAGCATT	259	Chicken IFN-β
chIFN-β-R	GGATGAGGCTGTGAGAGGAG		
chTNF-α-F	CCGCCCAGTTCAGATGAGTT	130	Chicken TNF-α
chTNF-α-R	GCAACAACCAGCTATGCACC		
ch-actin-F	GTCCACCGCAAATGCTTCTAAA	102	Chicken β-actin
ch-actin-R	CCATGCCAATCTCGTCTTGTTT		

### Transfection

Transient expression was achieved by transfection using Lipofectamine 2000 (Invitrogen), according to the manufacturer’s instructions. Briefly, cells were seeded in a 24-well plate one night prior to transfection. In total, 1 μg of plasmid(s) was mixed with 2 μl of liposome diluted in 50 μl of DMEM (without FBS or antibiotics) at room temperature. After 20-min incubation, the DNA and liposome mixture were added dropwise onto cells and incubated for 1 day for further analysis.

### Western Blot Analysis

Protein samples were separated by sodium dodecyl sulfate–polyacrylamide gel electrophoresis (SDS-PAGE) and then transferred to nitrocellulose (NC) paper (Bio-Rad). After being blocked in 5% skim milk, primary antibodies were incubated with NC paper at 4°C. On the following day, NC papers were washed with PBS with 0.05% Tween 20 (PBST) five times, followed by incubation with the corresponding secondary antibody conjugated to horseradish peroxidase (HRP) for 1 h. After being washed with PBST, protein signals were visualized by enhanced chemiluminescence and ImageQuant LAS 4000 (GE Healthcare, Uppsala, Sweden). The dilutions of each antibody were as follows: anti-β-actin (1:1,000; Signalway Antibody), anti-PKR (1:2,000; Abcam), anti-PKR-p (T446; 1:2,000; Abcam), anti-FLAG (1:2,500; Signalway Antibody), and anti-HA (1:2,500; Yao-Hong Biotechnology).

### Immunofluorescence Assay

As described previously (Liao et al., 2019), cells transiently expressing the target proteins were fixed with 1.875% formaldehyde, permeabilized with 0.5% NP-40, and then incubated with anti-NS1 (sc-130568, Santa Cruz Biotechnology) antibody or anti-NP antibody (ab20343, Abcam) for 1 h. Cells were further incubated with corresponding secondary antibodies conjugated with Alexa Fluor 488 (Invitrogen) for an additional 1 h at room temperature. Washing (PBS containing 1% FBS) was conducted between each step for a total of six washes. Subsequently, cells were treated with DAPI (4′,6-diamidino-2-phenylindole; Invitrogen) at a final concentration of 1 μg/ml for nucleic acid counterstaining. Images were acquired by confocal microscopy (FV1000, Olympus, Tokyo, Japan) with Olympus FV10-ASW 1.3 viewer software.

### Mini-Genome Reporter Assay

Briefly, 293T cells seeded in 48-well plates were co-transfected with a pcDNA3.1 plasmid expressing A/chicken/Taiwan/0702/2013 NP (20 ng), PB1 (20 ng), PB2 (20 ng), and PA (20 ng); a firefly luciferase expression plasmid (10 ng) as an internal control for transfection efficiency; and a reporter plasmid, pPol I-Flu-Rluc (100 ng) (Massin et al., 2005), containing ***Renilla*** luciferase of which the initial RNA transcription was controlled by the promoter of human RNA polymerase I. On the other hand, the expression of ***Renilla*** luciferase was driven by influenza polymerase complex that was kindly provided by Dr. Laurence Tiley at the University of Cambridge. At 24 hpi, the transfected cells were harvested, and luminescence was assayed using a Dual-Glo luciferase assay system following manufacturer instructions (Promega). Luciferase activity was then measured by a FLUOstar OPTIMA microplate reader (BMG Labtech GmbH, Offenburg, Germany). Relative light units (RLUs) were estimated as the ratio of ***Renilla*** to firefly luciferase luminescence. Relative luminescence was calculated as a percentage of the maximum RLUs in each experiment.

### Immunoprecipitation

The immunoprecipitation protocol was modified from a previous report (Liao et al., 2019). In brief, cells expressing NS1 tagged with FLAG were harvested in lysis buffer containing proteinase inhibitor cocktail (Roche Diagnostics GmbH, Mannheim, Germany). After clarification by centrifugation, one tenth of the whole-cell lysate (WCL) was kept as the input control. The remaining WCL was incubated with anti-FLAG M2 affinity gels (Sigma-Aldrich, St. Louis, MO, United States) at 4°C overnight. On the next day, the gel was washed 10 times with wash buffer [50 mM of Tris (pH 7.4) and 150 mM of NaCl]. Target proteins were then eluted in SDS sample dye and resolved by SDS-PAGE, followed by Western blot analysis.

### Statistical Analysis

The comparison of growth kinetics between each group and the parental virus was analyzed by repeated measures ANOVA, and the data were displayed as mean ± standard error. Differences among cytokine expression were evaluated by SAS (SAS Institute, Cary, NC, United States) using the Mann–Whitney *U* test, and data were displayed in box-and-whisker plots in GraphPad Prism 5 software (GraphPad Software, San Diego, CA, United States). *P*-values that are less than 0.05 were considered statistically significant.

## Results

### High Frequency of Sequence Variations in Avian Influenza Virus Non-structural Gene

**FIGURE 1:**
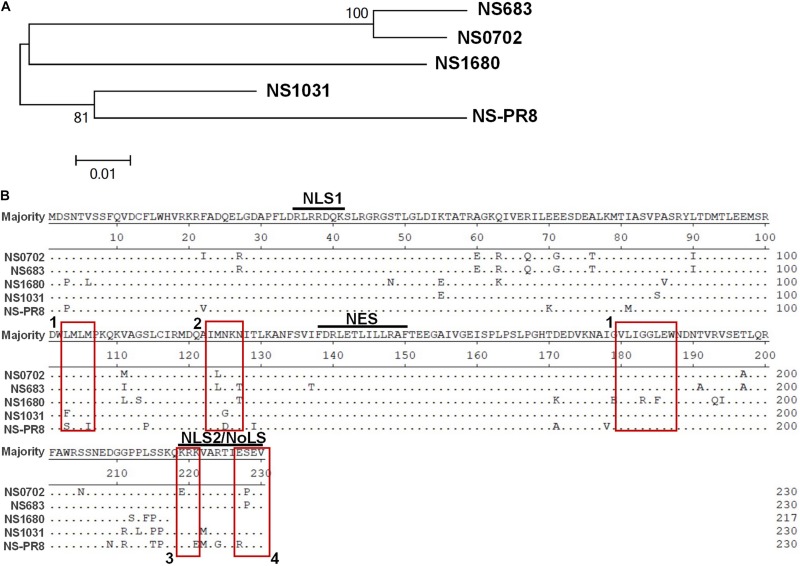
Sequence analysis of influenza virus non-structural protein 1 (NS1) proteins. **(A)** Phylogenetic analysis of NS1 proteins of several avian influenza viruses, including the NS1 from subtype H6N1 (NS0702), one LPAI H5N2 (NS683), two HPAI H5N2 (NS1680 and NS1031), and one human H1N1 (NS-PR8). **(B)** Sequence alignment indicated variations at residues responsible for interactions with cellular protein CPSF30 (box 1), protein kinase R (PKR) (box 2), SUMO-1 (box 3), and a PDZ-binding domain (box 4), as well as two sets of nuclear localization signals (NLS), the nucleolus localization signal (NoLS), and nuclear exportation signal (NES).

Since December 2003, the LPAI H5N2 has caused repeated epidemics in poultry farms in Taiwan. In 2012, the Mexican-like LPAI H5N2 evolved to HPAI. In early 2015, poultries in Taiwan suffered from infections of the clade 2.3.4.4 H5 HPAIVs, rather than the endemic Mexican-like H5N2 viruses. Sequence analysis revealed that the NS gene of these new viruses shared a distinct phylogenetic relationship with the endemic H6N1 and H5N2 viruses (Figure 1A and Supplementary Figure S1). Moreover, sequence alignment indicated that the similarity of the NS1 protein sequence between the four viruses was as low as 90.6% (A/chicken/TW/0702/2013 vs. A/chicken/TW/1680/2013), and the coding region of NS1680 was shorter than that of the other three NS1, namely, NS683, NS0702, and NS01031 (Figure 1B). Noticeably, sporadic variations were observed in the NS1 coding region; several of those occurred in key residues responsible for the interaction of NS1 with CPSF30 (Figure 1B, box 1) (Dankar et al., 2013), PKR (Figure 1B, box 2) (Min et al., 2007), SUMO-1 (Figure 1B, box 3) (Santos et al., 2013), and a PDZ-binding domain (Figure 1B, box 4) (Thomas et al., 2011), as defined based on sequences of strain PR8.

### Rescue and Characterization of Reassortant Viruses

To characterize the contribution of NS1 variants to viral replication, four reassortant H6N1 AIVs engineered by reverse genetics (RG-AIVs) were generated in the genetic background of A/chicken/Taiwan/0702/2013 (H6N1) carrying the NS segment of different H5 viral strains. The RG-AIVs were named according to the origin of the NS gene, that is, NS0702 (A/chicken/Taiwan/0702/2013), NS683 (A/chicken/Taiwan/683/2012), NS1680 (A/chicken/Taiwan/1680/2013), and NS1031 (A/goose/Taiwan/01031/2015). Of note, the four representative viruses were classified into distinct clades; eight genetic segments of NS0702 originated from the endemic H6N1 virus isolated in 2013, the same year as the HPAI H5N2 A/chicken/Taiwan/1680/2013 (NS1680); whereas A/chicken/Taiwan/683/2012 (NS683) and A/goose/Taiwan/01031/2015 (NS1031) originated from the endemic Mexican-like HPAIV H5N2 isolated in 2012 and clade 2.3.4.4 H5N2 isolated in 2015, respectively (Supplementary Figure S1). Each RG-AIV was purified to homogeneity and validated by automated sequencing.

**FIGURE 2:**
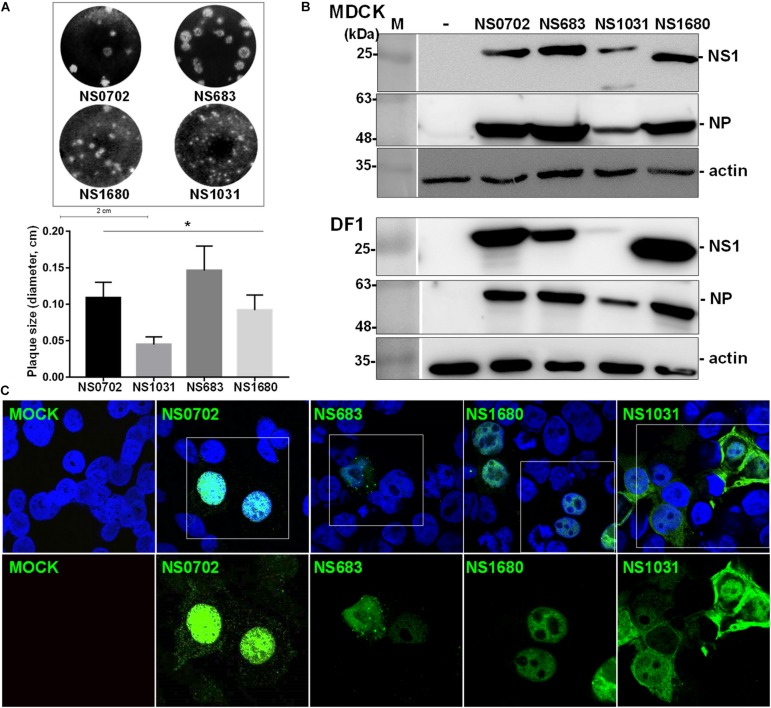
Characterization of recombinant avian influenza virus (AIVs). Four recombinant AIVs engineered by reverse genetics (RG-AIVs) containing eight gene segments from H6N1 (NS0702) or with substitution of the NS gene derived from other H5N2 viruses (NS683, NS1680, and NS1031) were rescued. **(A)** Plaque formation ability and average plaque size were indicated. **(B)** The expression level of NS1 and NP in mock (-) or infected Madin–Darby canine kidney (MDCK) or DF-1 cells was analyzed by Western blot. **(C)** Cellular distribution of NS1-eGFP enhanced green fluorescent protein in 293 cells was detected at 24 h after transfection by fluorescence microscopy, at 400× magnification. The image in white boxes on the top raw was further enlarged and shown in the lower panel. Nucleus was indicated in blue by DAPI staining. * indicates significant difference (*p* < 0.05) in plaque size relative to parental virus (NS0702).

Non-structural gene substitution affected plaque formation of RG-AIV on MDCK cells (Figure 2A). In particular, NS1031 plaques were significantly smaller than those of the other three RG-AIVs. Furthermore, the expression level of NS1 and NP proteins in NS1031 RG-AIV-infected MDCK and DF1 cells was lower than that of cells infected by the other three viruses (Figure 2B). As indicated in Figure 2C, NS1 was predominantly localized in the nucleus, except NS1031, the majority of which can be detected in nuclear, cytoplasm, or both nucleus and cytoplasm in 3, 18, and 31 of the 52 positive staining cells, respectively.

### Growth Kinetics of Avian Influenza Viruses Engineered by Reverse Genetics in Cells

**FIGURE 3:**
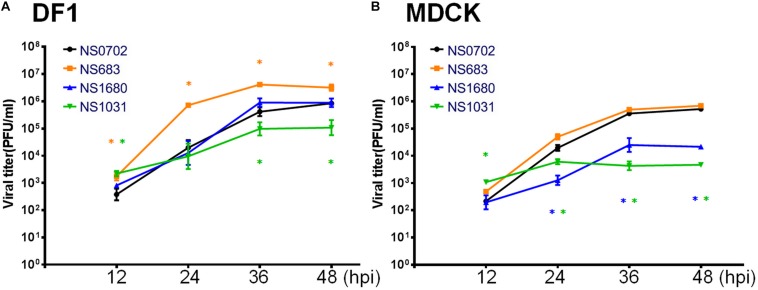
Growth kinetics of reassortant avian influenza virus (AIVs) in cells. **(A)** DF-1 or **(B)** Madin–Darby canine kidney (MDCK) cells were infected with one of four reassortant viruses at a 0.01 multiplicity of infection (MOI), and the yield of viral progeny was determined at 12, 24, 36, and 48 h post infection (hpi) by standard plaque assay [plaque-forming units (PFUs)/ml). The mean yields and standard error of triplicate experiments are displayed. *Indicates a significant difference (*p* < 0.05) between yield of the progeny and parental virus (NS0702).

Next, an analysis of RG-AIV growth kinetics was conducted in MDCK and DF-1 cells to evaluate the impact of NS gene substitution. The two types of cells were infected with each of the four reassortant viruses at a MOI of 0.01, and the yield of progeny virus was determined every 12 hpi (Figures 3A,B). In general, infection of all RG-AIVs was more efficient in DF-1 than MDCK, as indicated by the higher yield of viral progenies. Of note, infection of NS1031 in DF1 cells produced the lowest overall yield of viral progeny among the four RG-AIVs, significantly lower than NS0702 at later stages of infection (36 and 48 hpi) despite an initial significantly greater replication rate at 12 hpi (Figure 3A). NS 683, however, yielded significantly more progeny than NS0702 at all time points (Figure 3A). In MDCK cells, the yields of NS1680 and NS1031 progeny were significantly lower than those of parental NS0702 at later stages of infection (i.e., 24, 36, and 48 hpi; Figure 3B), and NS683 was not significantly different from NS0702 at any time point (Figure 3B). A consistent trend of viral yield was noted in human alveolar basal epithelial cells, A549 cells at 12 and 24 hpi, despite that overall the viral titer was lower than that produced in MDCK cells (Supplementary Figure S2).

### Detection of Cytokine Transcripts in Infected DF-1 Cells and Chickens

**FIGURE 4:**
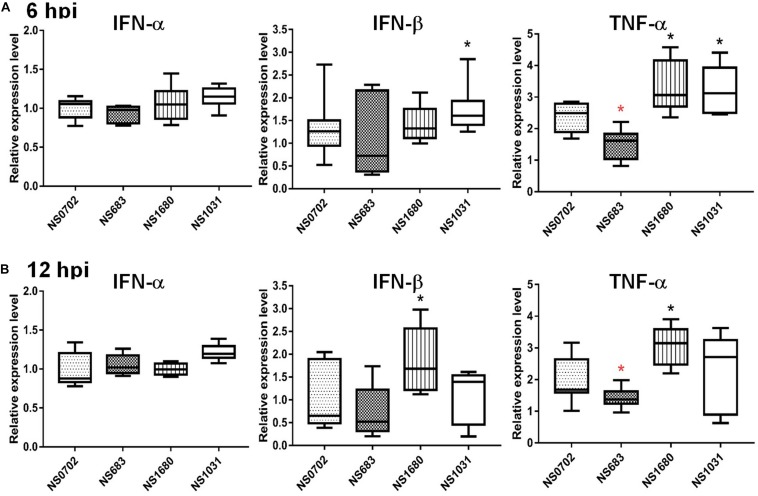
Expression of cytokines in DF-1 cells infected with reassortant avian influenza virus (AIVs). Chicken DF-1 cells were infected by reassortant AIVs at a multiplicity of infection (MOI) of 0.1. Total RNA was extracted at **(A)** 6 and **(B)** 12 hpi for detection of cytokine expression (IFN-α, IFN-β, and TNF-α) by qRT-PCR and normalized with mock infection. *Indicates significant difference (*p* < 0.05) in cytokine expression relative to parental virus (NS0702); black and red indicate significantly higher and lower expression, respectively.

The NS1 protein is well-known as an antagonist of type I IFN that mediates anti-viral responses. Therefore, we investigated whether the impact of NS substitution on viral production is related to the effect of NS1 on innate immunity. To do so, we measured transcripts of IFN-α/β and the pro-inflammatory cytokine tumor necrosis factor alpha (TNF-α), as well as viral M RNA in infected DF-1 cells or chicks by qRT-PCR at 6 and 12 hpi. In general, the viral M RNA was expressed to a similar extent among the viruses tested (Supplementary Figure S3). Relative to NS0702, NS1031 stimulated significantly higher levels of IFN-β and TNF-α expression at 6 hpi in DF-1 cells (Figure 4A). However, by 12 hpi, among all RG-AIV tested, only NS1680 stimulated higher expression levels of IFN-β and TNF-α than NS0702 (Figure 4B).

**FIGURE 5:**
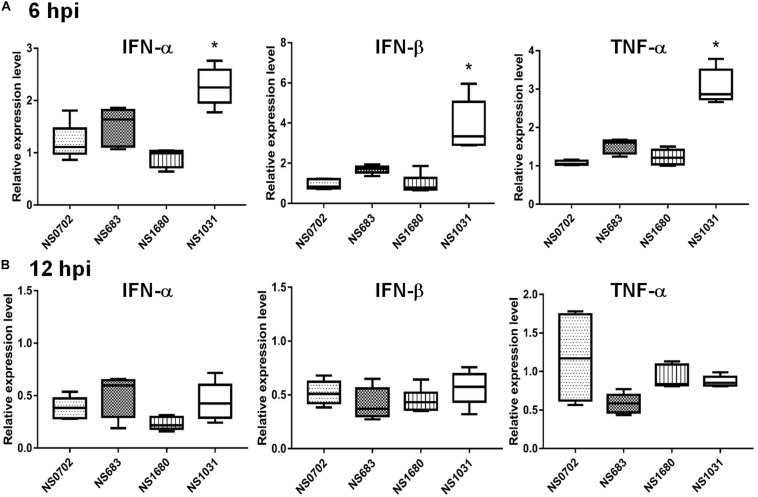
Expression of cytokines in chickens infected with reassortant avian influenza viruses engineered by reverse genetics (RG-AIVs). One-day-old chickens received AIV [5 × 10^5^ plaque-forming units (PFU)/chicken] by intratracheal instillation. Total IFN-α, IFN-β, and TNF-α RNA were extracted from the lungs at **(A)** 6 and **(B)** 12 hpi for qRT-PCR quantification of cytokines. *Indicates significant difference (*p* < 0.05) in cytokine expression relative to parental virus (NS0702).

Although IFN-α expression was not elicited by RG-AIV infections in DF1 cells relative to mock infection, it was significantly induced in chickens infected by NS1031. Furthermore, IFN-β and TNF-α were highly expressed in NS1031-infected chicken lungs at 6 hpi (Figure 5A), but this elevation of cytokine expression was diminished by 12 hpi (Figure 5B).

### Expression of IFN-α and TNF-α mRNA in Human Cells

**FIGURE 6:**
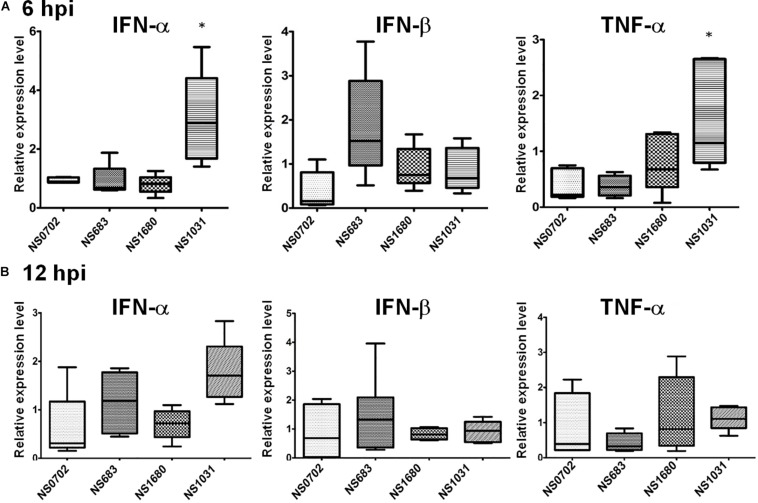
Expression of cytokines in human cells infected with reassortant avian influenza viruses (AIVs). Human A549 cells were infected by reassortant AIVs at a multiplicity of infection (MOI) of 0.1. Total IFN-α, IFN-β, and TNF-α RNA were extracted at **(A)** 6 and **(B)** 12 hpi, and cytokines were quantified by qRT-PCR.

Regulation of cytokine expression of RG-AIVs was also investigated in human cells. Human A549 cells were infected with reassortant viruses at a MOI of 0.1, and the expression levels of IFN-α and TNF-α mRNA were measured by qRT-PCR at 6 hpi. The results showed that cells infected with NS1031 expressed higher levels of IFN-α and TNF-α than those infected with parental virus (Figure 6A). However, no significant differences were observed at 12 hpi (Figure 6B).

### Effect of Non-structural Protein 1 of Avian Influenza on Protein Kinase R Activation

**FIGURE 7:**
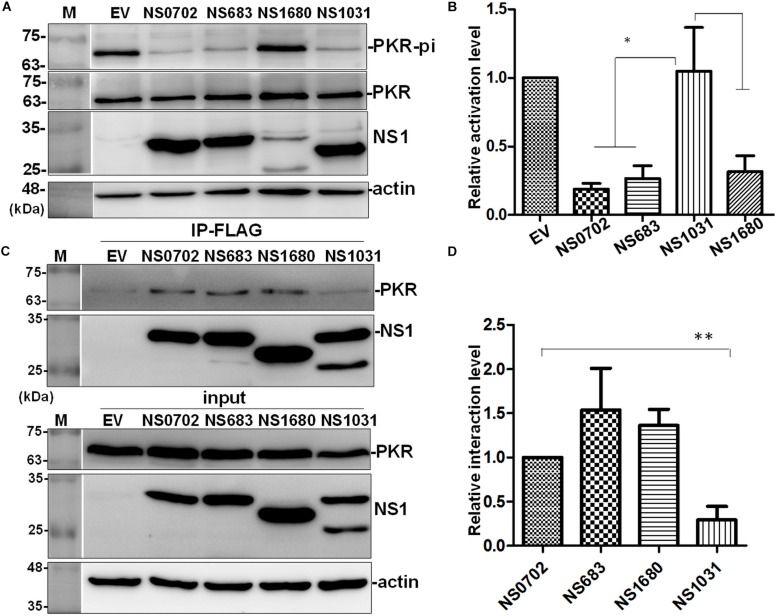
The effect of avian influenza virus (AIV) non-structural protein 1 (NS1) proteins on protein kinase R (PKR) activation and interaction. **(A,B)** Modulation of PKR activation by NS1. HEK293T cells were transfected with empty vector (EV) or plasmids expressing NS1 proteins, followed by activation of PKR by the transfection of 150 ng/ml of poly I:C for 4 h. The phosphorylation level of PKR was monitored by **(A)** Western blot analysis; **(B)** PKR activation level was estimated relative to EV. **(C,D)** Interaction of NS1 proteins with PKR. **(C)** HEK293T cells were transfected with plasmids expressing hemagglutinin (HA)-tagged PKR together with either EV or one of the NS1 constructs, followed by FLAG-IP and immunoblotting. **(D)** Relative PKR–NS1 interaction was estimated relative to EV. All experiments were conducted in triplicate. ^∗^ and ^∗∗^ indicate *p*-value <0.05 or <0.001, respectively.

Influenza NS1 is a well-known repressor of PKR activation by a direct interaction with PKR; however, the exact functions of these H5N2 NS1 proteins have not been investigated. In cells transiently expressing NS1, PKR activation was induced by treatment with synthetic dsRNA (poly I:C). NS1 of H1N1 (strain PR8) significantly decreased phosphorylation of PKR relative to empty vector (EV) (Figure 7A). Of note, among the four AIV NS1 proteins, only NS1031 failed to suppress PKR activation as evidenced by the high phosphorylation level. Moreover, results of immunoprecipitation suggested that NS1031 interacted poorly with PKR than did other AIV NS1 proteins analyzed and was significantly lower than NS0702 (Figure 7B).

### Effect of Non-structural Protein 1 on Viral Genome Activation

**FIGURE 8:**
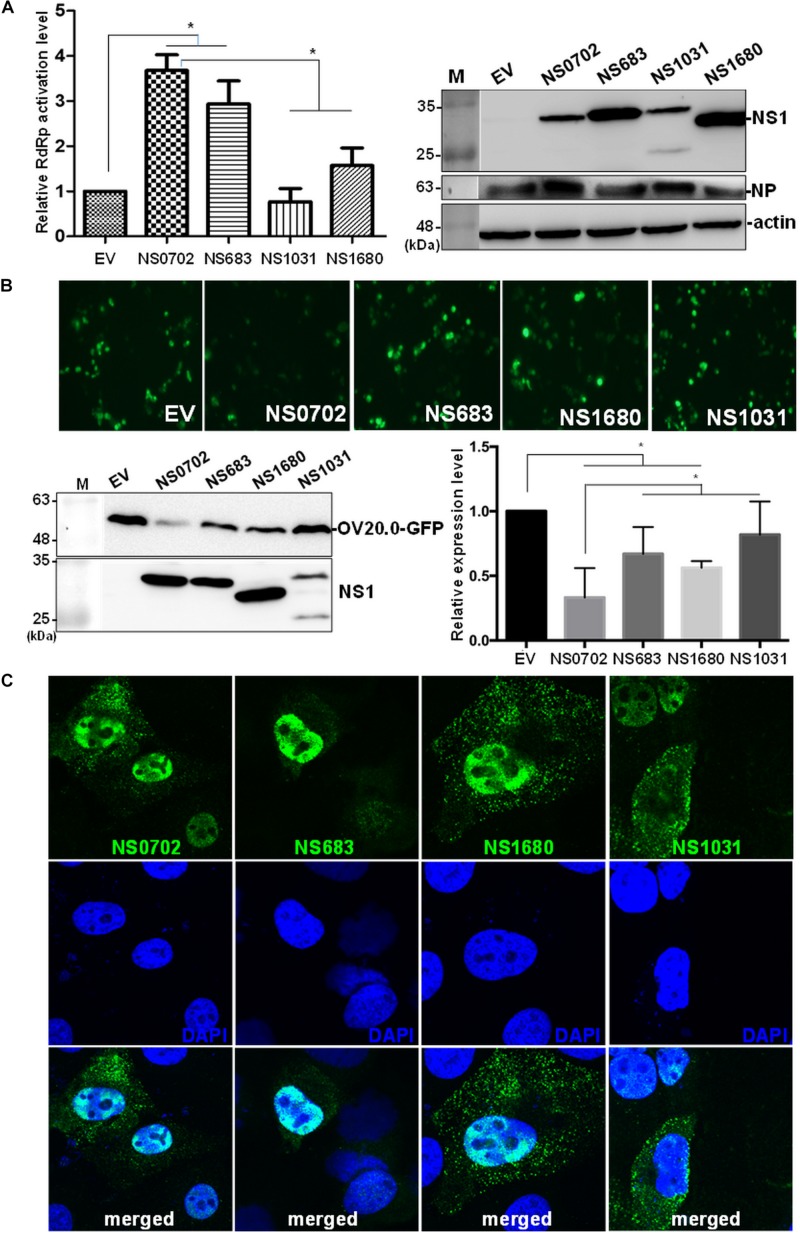
Impact of non-structural (NS) on viral genome activity. **(A)** Evaluation of the effect of non-structural protein 1 (NS1) on RNA-dependent RNA polymerase (RdRp) activity by mini-genome reporter assay. HEK293T cells were transfected with constructs expressing the viral RdRp complex (PA, PB1, PB2) and NP proteins, as well as a reporter plasmid pPol I-Flu-Rluc and internal control plasmid expressing firefly luciferase. At 24 h post transfection, luciferase activity and the expression of NS1 and NP of whole-cell lysate were monitored by luciferase assay and Western blot analysis, respectively. The Renilla luciferase expression level was initially normalized with firefly luciferase in the same group, and then the relative expression level was obtained by comparison with EV transfection, which was arbitrarily set as 1. The experiment was conducted in triplicate. *Indicates *p*-value <0.05. **(B)** Effect of NS1 on expression of a heterogeneous gene in cells. The individual NS1 was transiently co-expressed with a green fluorescent protein (GFP) fusion protein in HEK293T cells. Overall GFP signal was recorded by fluorescent microscopy (the top panel) and Western blot analysis (bottom, left panel); and the expression level, relative to that without NS1 protein, was measured and plotted (bottom, right panel). **(C)** Localization of ribonucleoprotein (RNP). Madin–Darby canine kidney (MDCK) cells were infected by individual avian influenza viruses engineered by reverse genetics (RG-AIVs) at a multiplicity of infection (MOI) of 1 for 24 h followed by immunofluorescent assay using antibodies against NP protein and DAPI to indicate the localization of RNP and the nucleus, respectively. The images were taken under confocal microscopy (400× magnification).

In addition to serving as an IFN antagonist, accumulating evidence indicates a modulatory role of NS1 on cellular and viral gene expression (Falcon et al., 2004; Wang et al., 2010; Kuo et al., 2016b). We exploited a mini-genome reporter assay to determine the possible role of NS1 on viral RdRp activity. Expression of NS0702 and NS683 proteins, as compared with EV, significantly enhanced viral genome replication activity, whereas NS1031 did not have a synergic effect on RdRp function (Figure 8A). Moreover, the effect of NS1 on general gene expression was also monitored by expression of GFP fusion protein driven by the CMV promoter (Tseng et al., 2015). As indicated in Figure 8B, NS0702 markedly reduced the GFP level, whereas NS1031 played no role on such an effect (Figure 8B). It was suggested that NS1 regulates gene expression *via* different mechanisms, which is likely by targeting the viral specific or cellular machinery.

### Effect of NS2 on Ribonucleoprotein Exportation

The NS gene encodes two proteins, namely, NS1 and NS2, by alternative splicing. As with NS1, sporadic sequence variations were noted in the NS2 coding region (Supplementary Figure S4). Nonetheless, the conserved residues responsible for the key function of NS2 (ref), including nuclear export signal, were conserved. NS2 facilitates the nucleocytoplasmic export of viral ribonucleoproteins (RNPs) (O’Neill et al., 1998; Shimizu et al., 2011); therefore, we investigated the NS2 function of these four RG-AIVs by monitoring the cellular localization of RNP. Although the majority of RNP remained in the nucleus, it also readily distributed to cytoplasm of cells infected with the four RG-AIVs, including NS1031, at 24 hpi (Figure 8C).

## Discussion

Different lineages of H5N2 AIVs emerged in Taiwan during the period 2003–2015, with each lineage showing distinct pathogenicity and high frequencies of sequence variations in the NS gene (Lee et al., 2014). The present study aimed to explore the relationship between the sequence divergence of the NS gene with viral pathogenicity. Our results revealed for the first time that substitution of NS gene affects viral progeny yield, cytokine modulation, and viral RdRp function.

The influenza virus NS1 protein is a determinant factor for viral replication and host adaptation, as well as for host anti-viral response counteraction (Seo et al., 2002; Hale et al., 2008; Rajsbaum et al., 2012; Abdelwhab et al., 2013). To investigate the potential impact of NS1 variations on contemporary AIVs, individual NS segments from three representative H5N2 strains were introduced into the backbone of an enzootic H6N1 virus by standard reverse genetics (Hoffmann et al., 2000). The reassortant viruses were propagated in SPF embryonic eggs followed by single-plaque purification. The titers of NS1031 virus were significantly lower than those of the other three RG-AIVs when initially amplified in embryonic eggs, and this pattern was mirrored in *in vitro* growth kinetic experiments, even at a late stage of infection (48 hpi). Moreover, among the four reassortant viruses, NS1031 gave rise to plaques with the smallest size. Given that plaque size generally represents the replication efficiency of influenza viruses (Ma et al., 2010), the low yield, retardation of growth kinetics, and decrease in plaque size indicated that NS1031 gene substitution leads to attenuation of the reassortant RG-AIV. Noticeably, cells that were infected with the NS1031 virus, as compared with NS0702, expressed significant higher levels of cytokines, including IFN-α, IFN-β, TNF-α; and that could be observed in both avian and human models (as summarized in Supplementary Table S1). Similarly, infection of the RG virus NS1680 stimulated a higher level of IFN-β and TNF-α in chicken DF1 cells, whereas cells infected with RG NS683 virus expressed a lower level of TNF-α in DF1 cells.

NS1 functions as a virulence factor for influenza viruses *via* interaction with different factors, exerting diverse functions to ensure efficient viral propagation. Among these, inhibiting both IFN production and counteracting the anti-viral effects of IFN-stimulated genes, namely, PKR and 2′5′-oligoadenylate synthetase (OAS)/RNase L, are well-described NS1-mediated actions that facilitate establishment at an early stage of infection (Hale et al., 2008). Noticeably, suppression of cytokine expression (IFN and TNF-α) was compromised in NS1031 RG-AIV at an early stage of infection (6 hpi), which is consistent with its defective inhibition of PKR activation (Figure 7), a factor potentially responsible for its poor infection outcomes (Figures 2A, 3). Although NS1 sequence polymorphisms are present at many of the signatures involved in such intermolecular interactions, including PKR (highlighted in Figure 1B, box 2), and previous studies have identified residues R35, R46, I123/M124, and K126/N127 as critical for NS1–PKR interaction (Min et al., 2007; Schierhorn et al., 2017), these residues are completely conserved in NS1031 (Figure 1B); thus, NS1 polymorphism cannot explain the low PKR association in NS1031. However, most research on key residues contributing to NS1 functionality has been conducted by a single amino acid change and loss-of-function studies, whereas reassortant viruses in our study possessed a high frequency of sequence polymorphisms spreading out over a large coding region of NS1 isolates, and, thus, it is likely that our local viruses possessed novel residues affecting PKR activation.

NS1 demonstrated a high frequency of variation at the C-terminus, not only in amino acid composition but also in length, among the four NS1 isolates analyzed in this study (Figure 1B); in particular, NS1680 carried a deletion of 218–230 aa. Residues 215–237 of NS1 interact with and impair the function of cellular poly(A)-binding protein II (PABP II), affecting maturation of pre-mRNAs, including IFN-β mRNA (Chen et al., 1999; Lin et al., 2007). We found that consistent with this knowledge, DF-1 cells infected with NS1680 had higher levels of IFN-β and TNF-α mRNA at 12 hpi. Moreover, four residues at the C-terminus of NS1, namely, PDZ-binding motifs (PBMs), have emerged as virulence determinants that modulate viral pathogenicity by mediating the association with cellular PDZ-domain-containing proteins that are involved in numerous cellular processes related to viral infection (Javier and Rice, 2011). Three sets of PBM sequences exist, with RSE/KV predominating the NS1 proteins of influenza viruses with human origins, whereas EPEV predominates in AIVs (Jackson et al., 2008), with the exception of ESEV, which predominates avian H5N1 virus isolates from human infections (Bao et al., 2007). Diverse PBM sequences were found in our four NS1 isolates; EPEV characterized both NS0702 and NS683, ESEV characterized NS1031, and NS1680 lacked PBM altogether. ESEV PBM is responsible for the interaction of H5N1 NS1 with cellular PDZ proteins, namely, Dlg1 and Scribble, which ultimately leads to disruption of the host tight junction and to increasing permeability of infected monolayers (Golebiewski et al., 2011); these pathologies are possible mechanisms of the severe disease associated with HPAIV H5N1. Taken together, it appears that substitution of the NS gene of AIV studied herein influences a spectrum of characteristics related to virulence and infectivity, and variations that occur at one particular known signature is unlikely to be fully responsible for the effect of an NS1 variant on countering PKR activation or modulating infection efficiency.

Of the three representative H5N2 strains, NS1031 gene is derived from the HPAI virus (A/goose/Taiwan/01031/2015). As NS1 is one of the virulent determinants, one might expect that the reassortant virus bearing NS1031 could possess a higher infection efficiency than the other recombinant viruses. Surprisingly, NS1031 gene substitution leads to attenuation of the reassortant RG-AIV. It has been shown that the HA gene of H5N2 virus emerging in 2015 was classified into the clade 2.3.4.4 of HPAIV, and with penta-basic-amino-acid HA0 cleavage site PLRERRRKR/GLF (Lee et al., 2016). Hence, it is likely that HA facilitates viral dissemination and is predominantly attributed to the high pathogenicity of the parental virus A/goose/Taiwan/01031/2015. On the other hand, substitution of NS gene might also render a poor coordination between NS1 with its viral counterparts, such as viral RdRp complex. It is well described that in addition to eliminating anti-viral responses, NS1 also modulates gene expression and viral genome activity *via* multiple mechanisms, including interaction with cellular machinery (Chen et al., 1999; Twu et al., 2007) or with the viral RdRp complex (Marion et al., 1997; Robb et al., 2011). Interestingly, we demonstrated *via* mini-genome assay that NS0702 and NS628 proteins significantly enhanced RdRp activity, whereas NS1031 did not contribute to viral genome activation (Figure 8A). Whether such a discrepancy is due to physical interaction of NS1 with RdRp is worthy of further investigation. Moreover, this result also indicated the possible genetic incompatibility of the reassortant NS1031 virus. The RG-AIV system in this study was based on an A/chicken/TW/0702/2013 (subtype H6N1) backbone; the internal genes of this H6N1 virus are similar to those of the Mexican-like H5N2 viruses, including A/chicken/TW/683/2013 and A/chicken/TW/683/2012 (Lee et al., 2014); whereas the clade 2.3.4.4 H5N2 virus, A/goose/TW/01031/2015, is genetically distant from A/chicken/TW/0702/2013 (Figure 1B), with internal genes reassorted from various AIV subtypes from different continents (Lee et al., 2016). Since 2015, these different lineages of H5 viruses and H6N1 virus have circulated the fields of Taiwan, but the reassorted viruses derived from clade 2.3.4.4 H5Nx viruses and the endemic Mexican-like H5N2 or H6N1 virus have not yet been identified. Mismatch between NS1 and the viral RNP complex composed of vRNA, RdRp, and NP might result not only in inefficient vRNA synthesis (Wang et al., 2010) but also in inadequate control of the host immune response in infected cells (White and Lowen, 2018). Therefore, it is possible that mismatch between the NS segment and viral RNP resulted in attenuation of NS1031 infections in this study, and these incompatible chimera viruses may not become a dominant type of virus in nature relative to their parental viruses.

## Conclusion

Our study extends the current understanding of AIV phylogenetic studies to characterize the NS1 function of H5N2 viruses isolated from Taiwan. We demonstrated that exchange of the NS segment significantly impacted the replication of reassortant viruses and modulated anti-viral responses and viral RdRp activity. These results indicate that NS1 is a critical factor responsible for the diverse traits of AIVs in Taiwan.

## Data Availability Statement

The datasets generated for this study are available on request to the corresponding author.

## Ethics Statement

The animal study was reviewed and approved by the Institutional Animal Care and Committee of National Chung Hsing University (approval IACUC number: 107-148).

## Author Contributions

W-LH and S-CO designed the experiment and analyzed the data. W-CW, C-YK, Y-JT, and C-HC conducted the experiments. Y-CL, Y-CC, and Y-CH performed the quantification of viral M gene by qRT-PCR and sequence alignment. M-KH and W-CW analyzed the data. W-LH wrote the manuscript.

## Conflict of Interest

The authors declare that the research was conducted in the absence of any commercial or financial relationships that could be construed as a potential conflict of interest.
