# Supplementary material for: The Impacts of Reassortant Avian Influenza H5N2 Virus NS1 Proteins on Viral Compatibility and Regulation of Immune Responses
Source: Front Microbiol. 2020 Mar 12;11:280. doi: 10.3389/fmicb.2020.00280 (PMC7080822; doi:10.3389/fmicb.2020.00280)
Supplement: Supplementary file 1 [file Data_Sheet_1.pdf]

Supplementary Material

Supplementary Table S1. Summary of cytokine levels in cells infected with different RG-AIVs.

		IFN-α		IFN-β		TNF-α	
		Low	High*	low	high*	low*	high*
DF1	6 hpi				NS1031	NS683	NS1031, NS1680
	12 hpi				NS1680	NS683	NS1680
Chicken	6 hpi				NS1031		NS1031
	12 hpi						
A549	6 hpi		NS1031				NS1031
	12 hpi						

Note: * indicated a significant difference in cytokine expression between the virus and NS0702.

Supplementary Figure S1

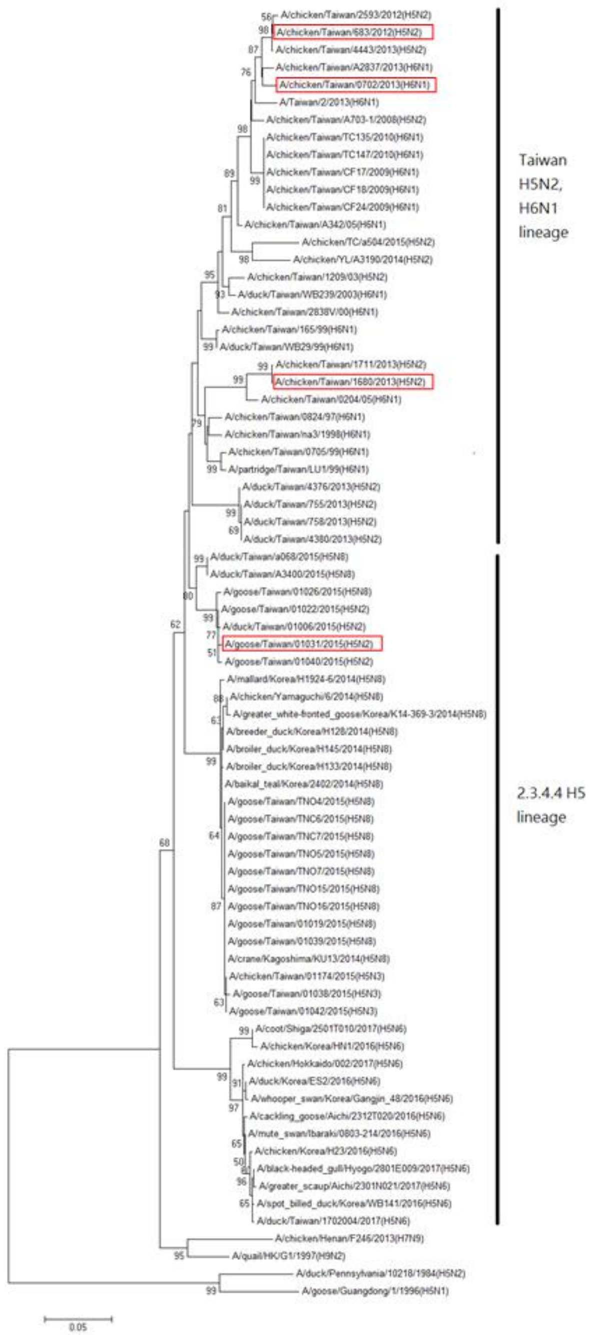

Supplementary Figure S1. Phylogenetic analysis of NS genes of influenza viruses. The phylogenetic tree was generated by the maximum-likelihood method with 1000 bootstraps replicates. The viruses isolated from Taiwan, including subtypes of H6N1, H5N2, were compared with those HPAI (2.3.4.4 lineage).

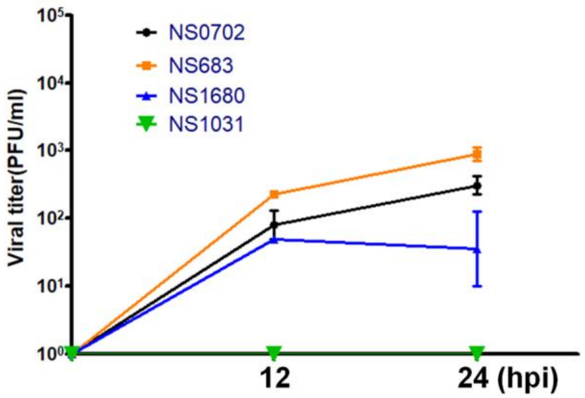

Supplementary Figure S2. Growth kinetics of reassortant AIVs in A549 cells. Human A549 cells were infected with each recombinant viruses at 0.01MOI. The yield of viral progenies were determined at 12, and 24hour post infection (hpi) by standard plaque assay. The experiment was performed in three individual repeats and the average yields were plotted.

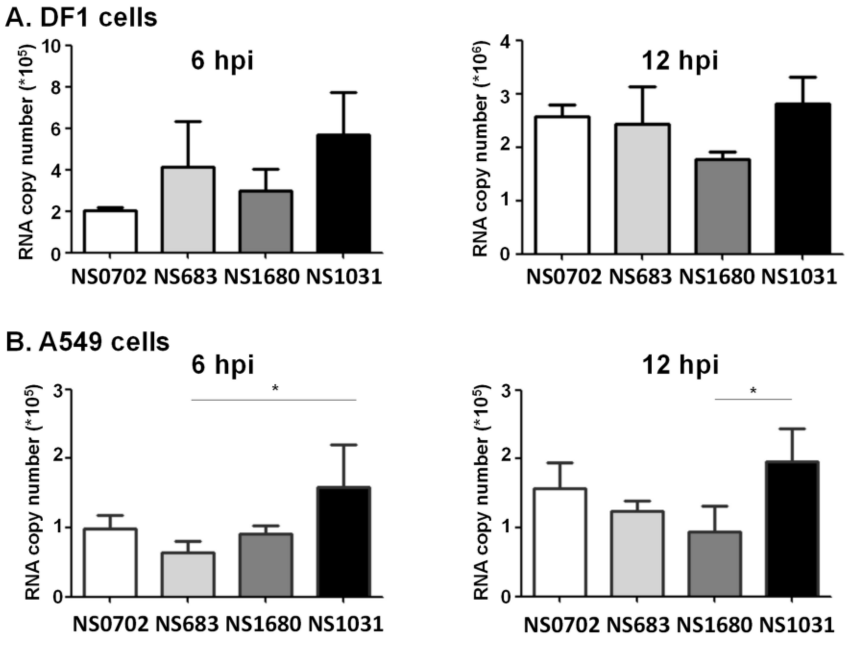

Supplementary Figure S3. RNA levels of viral M gene in DF1 or A549 cells infected with reassortant AIVs. Chicken DF1 (A), and human A549 cells (B) were infected by AIV at MOI of 0.1. Total RNA was extracted at 6 and 12 hpi for quantification of viral M RNA level by real-time PCR. Note * indicates significant difference ($p<0.05$) in copy number of M gene between the two viruses.

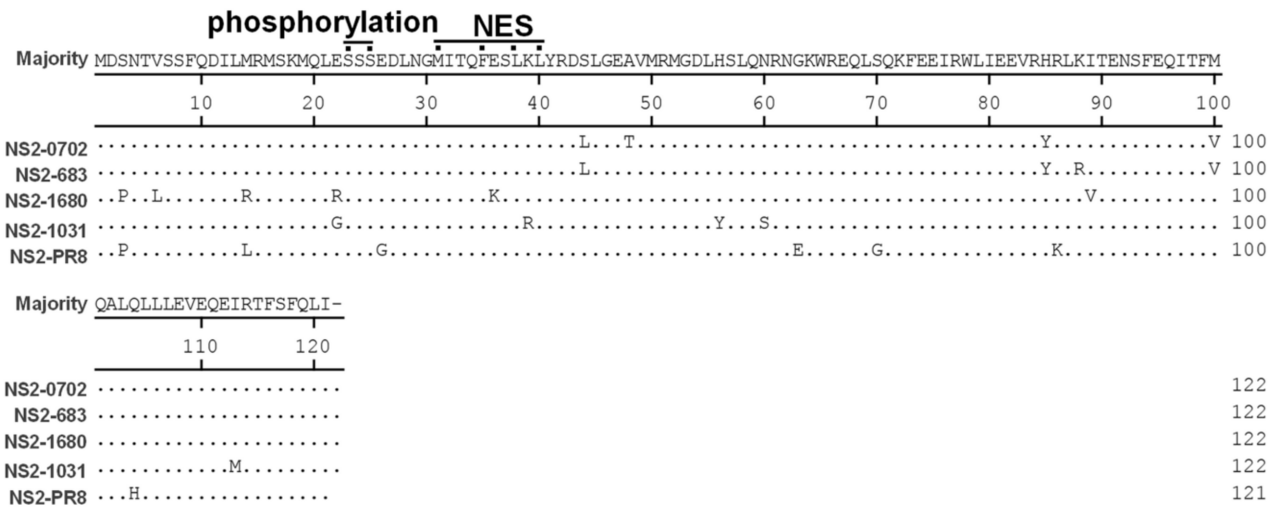

Supplementary Figure S4. Sequence analysis of influenza virus NS2 proteins. Sequences alignment indicates variations among the NS2 protein of the four avian influenza strains. The key residues (indicated as dots) for phosphorylation and nucleus exportation signal are highly conserved.
